# Clinical utility of long cap-assisted hemostasis in acute lower gastrointestinal bleeding: two cases

**DOI:** 10.1055/a-2724-7821

**Published:** 2025-11-06

**Authors:** Nobutaka Doba, Kosuke Shibayama, Shinzo Abe, Daiki Sakuma, Masanobu Someya, Kazuto Komatsu, Shin Maeda

**Affiliations:** 1 36998 Department of Gastroenterology, Yokosuka City Hospital, Yokosuka, Japan; 2 Department of Gastroenterology, Yokohama City University Graduate School of Medicine, Yokohama, Japan

Colonoscopy is essential for the diagnosis and treatment of lower gastrointestinal bleeding (LGIB) 1 . Cap-assisted colonoscopy improves visualization 2 , and long caps provide greater stability and a wider field of view compared with short caps ( Fig. 1 ). Their diagnostic utility in LGIB has been reported 2 3 , but video reports focusing on therapeutic advantages remain limited 4 5 . In this video, we present two cases demonstrating the effectiveness of this approach.

**Fig. 1:**
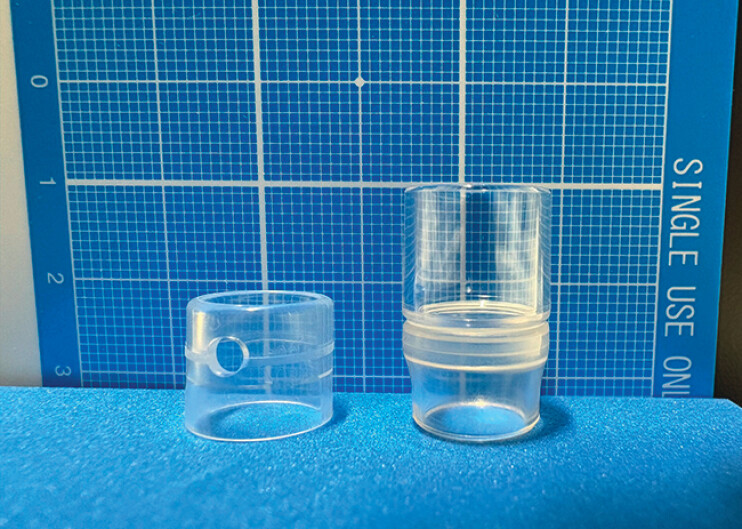
Comparison of cap types used in cap-assisted colonoscopy. Left: Short cap (D-201-13404, outer diameter: 14.0 mm, tip protrusion length: 4 mm, Olympus, Tokyo, Japan). Right: Long cap (MAJ-663, outer diameter: 15.8 mm, tip protrusion length: 12 mm, Olympus, Tokyo, Japan). The long cap offers an extended protrusion, providing a wider visual field and improved stability during endoscopic procedures compared to the short cap.

**Case 1** : A 79-year-old woman presented with fresh hematochezia. Computed tomography revealed fecal impaction in the rectum, raising suspicion of a rectal ulcer. Colonoscopy without bowel preparation showed the rectum filled with feces, and the bleeding source could not be identified ( Fig. 2 **a** ). Reinsertion with a long cap allowed fecal displacement and suction removal, enabling identification of an ulcer with an exposed vessel near the dentate line ( Fig. 2 **b** ). During preparation for hemostasis, spurting bleeding occurred, further impairing visualization ( Fig. 2 **c** ). Temporary hemostasis was achieved by compressing the vessel with the distal projection of the long cap, which reduced bleeding and secured a stable view ( Fig. 2 **d** ). Coagulation with hemostatic forceps was then performed successfully ( Video 1 ). Although tamponade can also be achieved with a short cap, its shorter tip restricts visualization and may create blind spots during active bleeding.

**Fig. 2:**
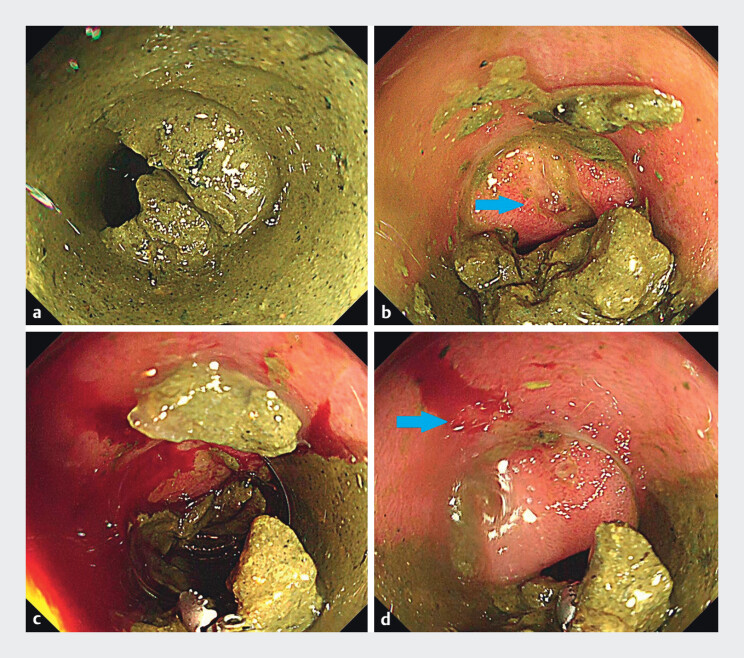
Endoscopic images from Case 1: rectal ulcer bleeding treated with long cap-assisted colonoscopy. **a** Pre-treatment image showing a large volume of feces in the rectum; the bleeding source could not be identified. **b** After fecal displacement and suction removal using the long cap, an ulcer with an exposed vessel near the dentate line was visualized (arrow indicates the exposed vessel). **c** During preparation for hemostasis, spurting bleeding occurred, obscuring identification of the exposed vessel. **d** The exposed vessel was clearly visualized and temporarily controlled by pressure hemostasis with the long cap (arrow indicates the exposed vessel).

Clinical utility of long cap-assisted hemostasis in acute lower gastrointestinal bleeding.Video 1

**Case 2** : A 77-year-old woman underwent colonoscopy for hematochezia, which revealed active bleeding from a sigmoid diverticulum ( Fig. 3 **a** ). By fitting the diverticulum inside the long cap, blood was displaced outside the visual field, enabling continuous observation ( Fig. 3 **b** ). This facilitated smooth clip deployment, achieving hemostasis ( Video 1 ). The larger inner lumen of the long cap preserved visualization without hindering device use.

**Fig. 3:**
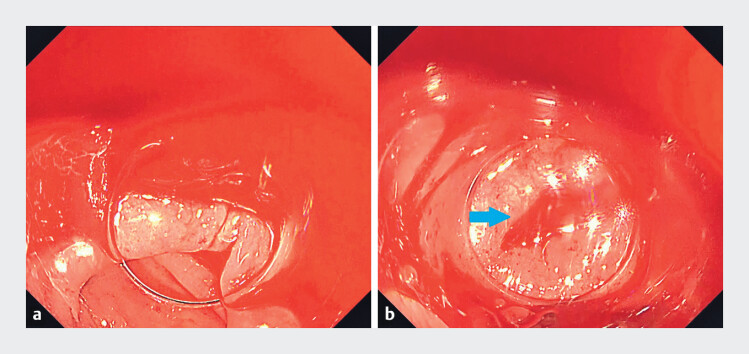
Endoscopic images from Case 2: sigmoid diverticular bleeding treated with long cap-assisted colonoscopy. **a** Pre-treatment endoscopic image showing a diverticulum with effusion bleeding; continuous visualization was difficult due to blood pooling and obscuration. **b** The responsible bleeding diverticulum positioned inside the cap; the long capʼs tip protrusion prevented blood from refluxing into the cap, enabling stable visualization (arrow indicates the responsible bleeding diverticulum). Abbreviation lower gastrointestinal bleeding: LGIB

Long cap-assisted colonoscopy not only secures visualization but also facilitates fecal and clot removal, tamponade, and stable device operation. It is a simple, low-cost, and effective accessory with potential for broader application in acute LGIB management.

Endoscopy_UCTN_Code_TTT_1AQ_2AZ

## Notes

Endoscopy E-Videos https://eref.thieme.de/e-videos
*E-Videos* is an open access online section of the journal *Endoscopy* , reporting on interesting cases and new techniques in gastroenterological endoscopy. All papers include a high-quality video and are published with a Creative Commons CC-BY license. Endoscopy E-Videos qualify for HINARI discounts and waivers and eligibility is automatically checked during the submission process. We grant 100% waivers to articles whose corresponding authors are based in Group A countries and 50% waivers to those who are based in Group B countries as classified by Research4Life (see: https://www.research4life.org/access/eligibility/ ). This section has its own submission website at https://mc.manuscriptcentral.com/e-videos .
